# Dietary Alterations in Impaired Mitochondrial Dynamics Due to Neurodegeneration

**DOI:** 10.3389/fnagi.2022.893018

**Published:** 2022-07-11

**Authors:** Ghulam Md Ashraf, Stylianos Chatzichronis, Athanasios Alexiou, Gazala Firdousi, Mohammad Amjad Kamal, Magdah Ganash

**Affiliations:** ^1^Pre-clinical Research Unit, King Fahd Medical Research Center, King Abdulaziz University, Jeddah, Saudi Arabia; ^2^Department of Medical Laboratory Technology, Faculty of Applied Medical Sciences, King Abdulaziz University, Jeddah, Saudi Arabia; ^3^Department of Science and Engineering, Novel Global Community Educational Foundation, Hebersham, NSW, Australia; ^4^AFNP Med Austria, Wien, Austria; ^5^Department of Health Sciences, Novel Global Community Educational Foundation, Hebersham, NSW, Australia; ^6^Institutes for Systems Genetics, Frontiers Science Center for Disease-Related Molecular Network, West China Hospital, Sichuan University, Chengdu, China; ^7^King Fahd Medical Research Center, King Abdulaziz University, Jeddah, Saudi Arabia; ^8^Department of Pharmacy, Faculty of Allied Health Sciences, Daffodil International University, Dhaka, Bangladesh; ^9^Enzymoics, Novel Global Community Educational Foundation, Hebersham, NSW, Australia; ^10^Department of Biology, Faculty of Science, King Abdulaziz University, Jeddah, Saudi Arabia

**Keywords:** Alzheimer’s disease, bayesian inference, dietary, mitochondrial dynamics, Python programming language, simulation

## Abstract

Alzheimer’s disease is still an incurable disease with significant social and economic impact globally. Nevertheless, newly FDA-approved drugs and non-pharmacological techniques may offer efficient disease treatments. Furthermore, it is widely accepted that early diagnosis or even prognosis of Alzheimer’s disease using advanced computational tools could offer a compelling alternative way of management. In addition, several studies have presented an insight into the role of mitochondrial dynamics in Alzheimer’s development. In combination with diverse dietary and obesity-related diseases, mitochondrial bioenergetics may be linked to neurodegeneration. Considering the probabilistic expectations of Alzheimer’s disease development or progression due to specific risk factors or biomarkers, we designed a Bayesian model to formulate the impact of diet-induced obesity with an impaired mitochondrial function and altered behavior. The applied probabilities are based on clinical trials globally and are continuously subject to updating and redefinition. The proposed multiparametric model combines various data types based on uniform probabilities. The program simulates all the variables with a uniform distribution in a sample of 1000 patients. First, the program initializes the variable age (30–95) and the four different diet types (“HFO_diet,” “Starvation,” “HL_diet,” “CR”) along with the factors that are related to prodromal or mixed AD (ATP, MFN1, MFN2, DRP1, FIS1, Diabetes, Oxidative_Stress, Hypertension, Obesity, Depression, and Physical_activity). Besides the known proteins related to mitochondrial dynamics, our model includes risk factors like Age, Hypertension, Oxidative Stress, Obesity, Depression, and Physical Activity, which are associated with Prodromal Alzheimer’s. The outcome is the disease progression probability corresponding to a random individual ID related to diet choices and mitochondrial dynamics parameters. The proposed model and the programming code are adjustable to different parameters and values. The program is coded and executed in Python and is fully and freely available for research purposes and testing the correlation between diet type and Alzheimer’s disease progression regarding various risk factors and biomarkers.

## Introduction

Mitochondria are vital organelles across every nucleated cell that generate energy in the form of ATP *via* the oxidative phosphorylation (OXPHOS) system (Saada, 2014). Mitochondria are defined as dynamic organelles that have been through coordinated cycles of fission and fusion, referred to as mitochondrial dynamics. Mitochondrial dynamics are ongoing processes of mitochondrial fusion, fission, biogenesis, and mitophagy that work together to maintain optimal cellular bioenergetics and reactive oxygen species (ROS) homeostasis (Archer, 2013). These mitochondrial dynamics regulate the number, distribution, and morphology of mitochondria in the cell and thus play critical roles in various mitochondrial functions such as energy production, metabolism, intracellular signaling, and apoptosis. Mitochondrial fusion is carried out by the dynamin-like GTPases regulatory mitofusin proteins mfn1, mfn2, and Opa1 (Itoh et al., 2013; Mishra and Chan, 2016). Mfn1 and Mfn2 are outer membranous proteins found on the outer side of the mitochondrial membrane, whereas OPA1 is a transmembrane protein found within the inner mitochondrial membrane (IMM) (Ni et al., 2015) that plays a role in mitochondrial quality regulation, which is mediated by mitophagy (Natarajan et al., 2020). Mitophagy corresponds to removing damaged or unnecessary mitochondria using autophagic machinery. Mitophagy is critical for mitochondrial quality control and homeostasis maintenance (Liao et al., 2017). Mitochondrial fission is required to form new mitochondria, excluding damaged mitochondria. Mitochondrial fission is essential for mitochondrial replication and the removal of damaged organelles *via* selective autophagy (Liao et al., 2017). The dynamin-related GTPase dynamin-related protein 1 (Drp1) is recruited by Fis1 protein from the cytosol to the mitochondrial outer membrane during mitochondrial fission (Cho et al., 2013). In contrast, mitochondrial fusion results in tubular or elongated mitochondria, which allow material exchange between mitochondria and can compensate for functional defects. Mfn1 and Mfn2 are responsible for mitochondrial outer membrane (MOM) fusion (Suárez-Rivero et al., 2017). OPA1 is responsible for mitochondrial inner membrane (MIM) fusion (Griparic et al., 2004; Suárez-Rivero et al., 2017). The OPA1 protein is involved in various functions, including respiratory chain and potential membrane maintenance, cristae organization and apoptosis control, and mtDNA maintenance (Griparic et al., 2004; Ni et al., 2015).

Mitochondrial morphology is dynamic and responsive to metabolic changes. Mitochondrial fusion is linked to increased ATP production, whereas fusion inhibition is linked to impaired OXPHOS, mtDNA depletion, and ROS production. OPA1 deficiency causes mitochondrial fragmentation and cell death in pancreatic cells, impairing insulin secretion and systemic glucose homeostasis. Alterations in mitochondrial dynamics can result in disease neuropathies characterized by impaired mitochondrial fusion and transport or significant optic atrophy caused by reduced mitochondrial fusion (Züchner et al., 2004). Mitochondrial dynamics shed new light on the pathophysiology of mitochondrial disorders and other diseases associated with mitochondrial dysfunction, such as diabetes, heart failure, and neurodegenerative diseases like Alzheimer’s disease (AD) (Alexiou et al., 2018a,b).

Additionally, different dietary elements have been suggested to affect AD and mitochondrial function and their dynamic behavior, such as Omega 3 (PUFA), Ketogenic Diet, and saturated fatty acid. Increased fat oxidation, energy expenditure, and reduced-fat deposition are potential effects of omega 3 PUFAs that could help prevent obesity and related metabolic disorders (DeFronzoand Tripathy, 2009). The capacity of omega 3 polyunsaturated fatty acids (PUFAs) to reduce inflammation, a characteristic of obesity and related metabolic disorders is well established (Buckley and Howe, 2010; Lalia and Lanza, 2016; Lepretti et al., 2018). Insulin resistance is closely linked to the emergence of inflammatory pathways, mitochondrial dysfunction, oxidative damage, and ER stress. The positive modulatory effects of omega 3 PUFAs on Mfn2 may explain the improvement of mitochondrial performance and the stimulation of fusion events related to maintaining the mitochondrial-associated endoplasmic reticulum membrane integrity, given Mfn2’s unique position as an ER-mitochondria bridge (Lepretti et al., 2018). Amyloid plaques, including beta-amyloid peptides (A-βeta), are the neuropathological hallmarks of AD (Mattson, 2004). According to recent studies, soluble oligomeric forms of Aβ may play a vital role in the etiology of AD. Amyloid Precursor Protein Processing (APP) is inextricably linked to cellular membranes, and membrane biophysical characteristics are crucial (Chen et al., 2017). PUFAs play an essential role in defining cell membrane fluidity. There is evidence that membrane fluidity influences APP processing regulation in multiple ways (Afshordel et al., 2015; Gammone et al., 2019), while A-βeta reduces membrane fluidity and thus stimulates its production, starting a vicious cycle (Pagani and Eckert, 2011). Recent *in vitro* findings suggest that the positive effects of docosahexaenoic acid (DHA), found in fish oil, are linked to mitochondria and APP processing modulation and significantly impact mitochondrial membrane phospholipid composition and function (Chen et al., 2017).

Modern biodata is characterized by abundance and diversity, including nucleic acid structures, gene expression levels, molecular interactions maps, gene maps, prediction of proteins’ unfolding, mutants identification, and pattern and motifs recognition mainly based on mathematical and computational models of low complexity and high efficiency. Mathematical Biology includes many interdisciplinary fields applied to modeling a natural phenomenon or mechanism, such as Theory of Algorithms, Data Mining, Genetic Algorithms, Neural Networks, Artificial Intelligence, and Machine Learning, Combined Learning Mathematics, Bayesian Statistics, Stochastic Analysis, Pattern Recognition, and Simulation. Theoretical models are a set of rules and laws that represent this phenomenon. When these rules or laws are expressed in mathematical relations, we refer to a mathematical model, a structure that approaches the properties of a random phenomenon through a simplification process. Mathematical modeling enables the analysis and monitoring of complex biological processes revealing any possible connections or disruptions that may lead, for example, to the development of a hypothesis related to a disease etiology or a drug discovery (Fischer, 2008). However, sometimes the limitations in the accuracy and the complexity of a mathematical model in Biology may lead to insufficient clinical application. Therefore, in this study, we formulate the probabilistic expectations of AD development or progression due to specific risk factors or biomarkers related to the impact of diet-induced obesity with an impaired mitochondrial function and altered behavior. We designed and programmed a Bayesian model to formulate and test diet type and AD progression correlation regarding various risk factors and biomarkers.

## The Ketogenic Diet Offers a Preventive Ability for Alzheimer’s Disease

In ketogenic diets (KD), fats are in huge quantity, proteins are comparatively low, and carbohydrates are in insufficient quantity, which results in protein and carbohydrates’ limited metabolism and the metabolic rate of fats being high (Allen et al., 2014; Lima et al., 2014). The ketogenic diet is used to treat neurodegenerative diseases such as AD because ketone bodies such as acetoacetate (AA) and -hydroxybutyrate (-OHB) reduce oxidative stress and improve mitochondrial biogenesis and function (Branco et al., 2016). The ketogenic diet makes it a viable alternative energy precursor. Furthermore, this diet may aid in the reduction of amyloid plaque accumulation as well as the reversal of amyloid-beta toxicity (Broom et al., 2019). KD may address these metabolic issues while protecting against A plaques associated with AD. Recent memory loss is associated with amyloid-βeta (Aβ) peptide deposition and hippocampal neuronal death in AD. *In vitro* studies suggest that the ketogenic diet can help with this, as -OHB has been shown to protect against toxicity in cultured hippocampal neurons (Branco et al., 2016). Evidence suggests that brain ketone uptake is not impaired in AD, unlike glucose.

## Influence of Dietary Saturated Fatty Acid on Mitochondrial Dynamics

Different fat sources (high lard diet) in a high-fat diet affected serum levels of metabolites associated with the development of obesity and obesity-related diseases differently. A high-fat, saturated-fatty-acid-rich diet (high lard, HL, diet) resulted in hepatic fat accumulation and insulin resistance and impaired mitochondrial function, increased ROS production, and decreased production of mitochondrial motility proteins (Lionetti et al., 2014; Putti et al., 2015; Chen et al., 2018). Mfn2 expression was reduced in the skeletal muscles of obese Zucker rats and type 2 diabetic patients (Bach et al., 2005; Hernández-Alvarez et al., 2010; Putti et al., 2015). Besides that, saturated fatty acids have been shown *in vitro* to induce fission processes in differentiated C2C12 skeletal muscle cells, associated with mitochondrial dysfunction (Jheng et al., 2012). *In vivo*, smaller mitochondria and increased mitochondrial fission machinery have been observed in the skeletal muscles of genetically obese rats and those with diet-induced obesity, as previously described (Jheng et al., 2012). Rats fed high-lard (L rats) and high-fish-oil (F rats) diets presented similar increases in energy intake in comparison with the intake of rats fed standard diets (N rats) (Lionetti et al., 2014). L rats significantly gained additional bodyweight than F rats (Lionetti et al., 2014). Serum TG and ALT levels were significantly higher in L rats than in N rats and both parameters were lower in F rats than in L rats, with TG levels not differing between F and N rats (Lionetti et al., 2014). L and F rats also displayed significantly higher serum glucose levels than N rats. In contrast, the serum insulin level and the HOMA index value were highest in L rats, with TG levels not differing between F and N rats (Lionetti et al., 2014). Compared to high lard feeding, high fish oil feeding was associated with the development of obesity, dyslipidemia, insulin resistance, and a lower degree of liver injury. Protection against ROS damage through mild uncoupling markers of mitochondrial oxidative stress was evaluated (Lionetti et al., 2014). Because once compared to both N and F rats, L rats generated more mitochondrial ROS due to their lower basal/total aconitase activity ratio. Similarly, to their significantly higher H_2_O_2_ production in isolated mitochondria. The administration of a high-fat diet to rats increases the activity of AgRP neurons and significantly disrupts systemic energy metabolism (Figlewicz et al., 2013). Mfn2 deletion in these neurons prevents these adverse metabolic effects: fat mass was reduced, insulin and glucose levels were legitimized, and obesity was avoided (Dietrich et al., 2013). A high-fat diet caused mitochondrial fragmentation in AgRP neurons, leading to the hypothesis that reduced mitochondrial fusion contributes to neuronal regulation of whole-body energy metabolism and behavior patterns. A high-fat diet suppresses POMC neurons, which produce various peptide hormones that reduce appetite, food intake, and body weight (Wai and Langer, 2016). Unlike the effects in AgRP neurons, genetic ablation of Mfn2 in POMC neurons results in severe obesity characterized by overeating, low energy expenditure, and endocrine dysregulation. Mfn2 POMC deletion did not disrupt whole-body energy homeostasis, whereas Mfn1 POMC deletion did.

## Mitochondrial Dynamics in Alzheimer’s Disease

The fusion events are significant because they enable mitochondria to mix their contents, facilitating many vital functions like an equal distribution of metabolites, mtDNA repair, protein complementation, autophagy promotion, and isolation of damaged mitochondrial segments (Twig et al., 2008). On the other hand, the fission events enhance mitochondrial distribution along the cytoskeletal tracks and divide mitochondria equally into two daughter cells. Any malfunctioning in this process may result in programmed cell death. Fusion and fission, motility, transport, and mitophagy consist of the other three essential aspects of mitochondrial dynamics. These points are especially critical in neurons as they require mitochondria at the sites far from the cell body, and they are essential for functioning in cells of smaller size. A significant decrease in the mitochondrial movement has been reported when there are defects in fusion and fission. The decline in brain mitochondria’ normal functioning with increasing age has been associated with increased mitochondrial biogenesis (Grimm and Eckert, 2017).

Neurodegenerative diseases are observed to be associated with mitochondrial dysfunction, illustrating the importance of mitochondrial dynamics for human health. Mitochondria’s fusion and fission process maintain significant mitochondrial from and integrity. So, dysfunctions in mitochondrial fusion or fission may also contribute to neurological disorders. Motor neuron diseases characterized by progressive axonal degeneration have altered mitochondrial transport through the cytoskeleton. Mitochondria must be adequately positioned to meet the demands of the cell. Accordingly, mitochondria are delivered to areas of the axon where metabolic demand is high, such as synapses, active cones, and branches, or areas where axonal protein synthesis occurs (Babic et al., 2015). Alzheimer’s disease is one example of how mitochondrial transport has been linked to pathophysiological alterations (Magrané et al., 2014). In neurons, mitochondrial fission and fusion are essential for mitochondrial transport (Sheng and Cai, 2012). Fission is affected by Drp1 inhibition because it leads to enlarged mitochondria that cannot be adequately localized to dendrites or axons, and it suppresses synaptic formation and function (Chen et al., 2007; Kageyama et al., 2012; Itoh et al., 2013).

Mitophagy defects can cause the mitochondrial respiratory function to be lost in Drp1-null neurons, and Drp1 may be deficient in HeLa cells due to oxidative damage to mitochondrial components. ROS generation can be exacerbated by impaired respiration (Kageyama et al., 2012; Itoh et al., 2013). Although the processes underlying these alterations are unknown, there could be direct consequences of amyloid-beta (Ab), as AB fragments increased in mitochondria and mitochondrial mass decreased in cultured neurons’ neurites. S-nitrosylation-mediated enhancement of Drp1 activity is required for this mechanism (Cho et al., 2009). Mitochondrial morphological abnormalities, displayed as fragmented mitochondria with damaged inner membrane structures, have been progressively observed in neurons from AD patients and AD animal models overexpressed or treated with Aβ or tau (DuBoff et al., 2012; Manczak and Reddy, 2012; Li et al., 2016). Outward deposition of amyloid plaques is followed by accumulation of intraneuronal neurofibrillary tangles of hyperphosphorylated tau on a pathological level. It is linked to the loss of synapses, which leads to neuronal death (Huang and Mucke, 2012).

The lack of mitochondria or fusion and fission regulators in neurites and concurrent abnormalities in axon repair and axonal transport implies that mitochondrial and neuronal dysfunction in AD may be ascribed to mitochondrial trafficking impairment (Gao et al., 2017). Nonetheless, faulty mitochondrial morphology and transport in cell bodies, axons, and synaptic terminals produce localized energy in AD. Deficiency causes or exacerbates neuron malfunction and destruction in AD. As a result, aberrant mitochondrial dynamics could be critical in mitochondrial malfunction and neuronal dysfunction in the AD brain (Huang and Mucke, 2012).

## Materials and Methods

### Experimental Method

In this research paper, a new mathematical model is designed to formulate the correlation of AD progression with diverse diets and its mitochondrial dynamics side effects. The computational implementation was made in the Python programming language, and the Bayesian inference was applied in simulating data.

Bayesian theory is based on probability theory. Even though many supporters of the Classical approach oppose the Bayesian Inference due to the weak approach of prior distributions, the Markov Chain Monte Carlo theory was provided as a solution for this problem for disease assessments with satisfactory results (Tzoufras, 2009). Bayesian statistics uses random variables to pre-define the prior distribution of the desired model and calculate the posterior distribution f(θ | y). This posterior distribution can be expressed as (Vidakovic, 2011; Højsgaard, 2012):


(1) $$\text{f}\,\left(\theta |\text{y}\right)=\frac{\text{f}\,\left(\text{y}|\theta \right)\,\text{f}\,\left(\theta \right)}{\text{f}\,\left(\text{y}\right)}\propto \text{f}\,\left(\text{y}|\theta \right)\,\text{f}\,\left(\theta \right),$$

including both the prior and the observed data by the expression of the prior distribution f(θ) and the likelihood f(y | θ) (Alexiou et al., 2017) as follows:


(2) $$\text{f}\,\left(\text{y}|\theta \right)=\prod_{\text{i}=1}^{\text{n}}\text{f}\,\left({\text{y}}_{\text{i}}|\theta \right),$$

Considering the probabilistic expectations of AD development or progression due to specific risk factors or biomarkers (Table 1), we designed a Bayesian model to formulate the impact of diet-induced obesity with an impaired mitochondrial function and altered behavior (Putti et al., 2015; Alexiou et al., 2017). The applied probabilities are based on clinical trials globally and are continuously subject to updating and redefinition (Alexiou et al., 2017). Therefore the proposed model and the programming code are adjustable to different parameters and values.

**TABLE 1:** The relevant probabilities affecting Alzheimer’s disease progression related to age and factors that influence Obesity and Mitochondrial Dynamics, according to the literature (Christen, 2000; Modrego and Ferrández, 2004; Wang et al., 2009; Israeli-Korn et al., 2010; Barnes and Yaffe, 2011; Alzheimer’s Association, 2015; Alexiou et al., 2017; Mantzavinos and Alexiou, 2017).

Biomarker	Relevant probability affecting AD progression
Age (> 85)	38%
Age (75–84)	43%
Age (65–74)	15%
Age (< 65)	4%
Hypertension	∼20%
Oxidative Stress	25–30%
Obesity	3.4%
Depression	13.2%
Physical Activity	17.7%
DRP1	74.3%
OPA1	61.4%
MFN1	27.8%
MFN2	33.6%
FIS1	60%

**FIGURE 1:**
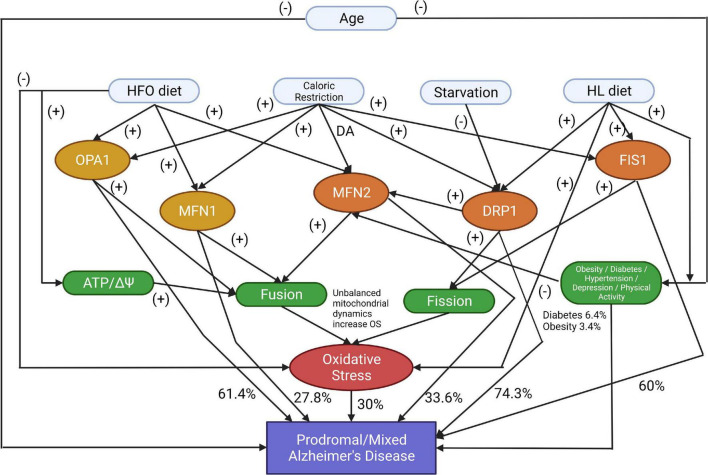
The proposed model identifies the markers that affect Prodromal or Mixed Alzheimer’s disease development or progression (DA, Don’t Affect; HFO diet, a high-fat diet rich in fish oil; HL diet, a high-fat diet rich in saturated fatty acids). The symbol (-) shows that the parent node increases the possibility of the child node occurring. The symbol (+) shows that the current node is a deterrent factor for activating the next node.


The proposed Bayesian model is presented in an acyclic graph (Figure 1), and the related variables are formulated using the uniform distribution:

(3) $$p\,\left(x|\alpha \right)∼U\,\left(0,\alpha \right)=\left\{ \begin{array}{cc} \frac{1}{a}\; & i\,f\,0\leq x\leq a\\ 0\; & o\,t\,h\,e\,r\,w\,i\,s\,e \end{array}\right.$$

For the Bayesian inference, we mainly apply a conjugate prior in medical experiments, where this conjugate prior to the uniform distribution is the Pareto distribution (Tenenbaum, 1998):


(4) $$p\,\left(a\right)∼P\,a\,\left(b,K\right)=\left\{ \begin{array}{cc} \frac{K\,b^{K}}{a^{K+1}}\; & i\,f\,a\geq b\\ 0\; & o\,t\,h\,e\,r\,w\,i\,s\,e \end{array}\right.$$

## Results

The program is coded and executed in Python and is fully and freely available for research purposes and testing the correlation between diet type and AD progression regarding various risk factors and biomarkers. The proposed multiparametric model combines various data types based on uniform probabilities. The calculated error is the Monte Carlo Error that measures the variability of each estimation due to simulation, increasing the model’s accuracy almost to 100% (Alexiou et al., 2017). Furthermore, in this Bayesian model, every biomarker (knots in the graph) can be linked with more than one other biomarker in the form of False | False, False | True, True | False, True | True (Alexiou et al., 2017).

The program simulates all the variables with a uniform distribution in a sample of 1,000 patients. First, the program initializes age (30–95) and the four different diet types (“HFO_diet,” “Starvation,” “HL_diet,” “CR”) along with the factors that are related to prodromal or mixed AD (ATP, MFN1, MFN2, DRP1, FIS1, Diabetes, Oxidative_Stress, Hypertension, Obesity, Depression, and Physical_activity). Then, the program executes the simulation according to the probabilities of Table 1, assigning values 1 or 0 in the case of a positive (abnormal) or not positive biomarker. The outcome is a table with the probability of AD development or progression related to diet choices and mitochondrial dynamics parameters (Table 2).

**TABLE 2:** Randomly selected results from the simulation of the 1,000 patients, in accordance with the five cases above.

Case	atp	opa1	mfn1	mfn2	drp1	fis1	db	os	ht	ob	dp	pa	diet	age	fusion	fission	AD
1	0	0	0	0	0	0	1	0	1	0	1	0	HFO	63	0	0	0.2
2	1	1	1	1	0	1	1	0	0	1	0	1	HL	31	1	1	0.614
3	1	0	1	0	0	0	0	0	0	1	1	0	starv	70	0	0	0.278
4	1	1	0	1	0	1	0	0	1	0	1	1	CR	86	1	1	0.614
5	0	0	0	0	0	0	1	0	1	0	0	0	0	80	0	0	0.743

*The probabilities refer to the development or progression of Prodromal/Mixed AD as defined by the “Research criteria for diagnosing AD: revising the NINCDS-ADRDA criteria” (Dubois et al., 2007). Keys: [atp, adenosine triphosphate; opa1, optic atrophy-1; mfn1, mitofusin-1; mfn2, mitofusin-2; drp1, dynamin-related protein; fis1, mitochondrial fission 1; db, diabetes; os, oxidative stress; ht, hypertension; ob, obesity; dp, depression; pa, physical activity; diet (type), starv (starvation); high-fat diet rich in fish oil (HFO), high-fat diet rich in saturated fatty acids (HL), Caloric Restriction (CR)], fusion and fission corresponds to the mitochondrial dynamics and AD to AD.*

The model represents specific proteins or diets’ negative or positive impact on developing AD. The symbol (-) shows that the parent node increases the possibility of the child node occurring. The symbol (+) shows that the current node is a deterrent factor for activating the next node. In the experimentation, zeros and ones were used as the result of an activation function that checks if the node’s current state is efficient enough to activate the next node. These calculations have included all the factors, with corresponding prior probabilities applied to uniformed distributed data. Results show different combinations of those factors that produce specific patients’ profiles and cases. Besides the known proteins related to mitochondrial dynamics, our model includes risk factors like Age, Hypertension, Oxidative Stress, Obesity, Depression, and Physical Activity, which are associated with Prodromal AD.

Strong evidence suggests the necessity of an optimized bioenergetics balance between mitochondrial fusion and fission (Putti et al., 2015). Therefore we present a few cases generated from our simulation accordingly to the corresponding theory and the relevant probabilities affecting AD progression related to age and other factors (Table 1).

**Case 1**: Subject with decreased oxidative stress due to HFO diets resulting in increased mfn2 expression and ATP levels and promoting fusion within a risk group due to the age (age > 60):


P(ProdromalMixedAD|HFO)=P(ProdromalMixed AD|HFO)P(ProdromalMixed)P(HFO) =0.2

**Case 2**: Subject with abnormal mitochondrial fission proteins (fis1 increased) and mfn2 (decreased) associated with high fat diet-induced obesity (HL diet), leading to an increased ROS:


P(ProdromalMixedAD|HL)=P(ProdromalMixed AD|HL)P(ProdromalMixed)P(HL) =0.614

**Case 3**: Subject with increased mfn1 and opa1 due to starvation-induced mitochondrial fusion, which increases ATP, within a risk group due to the age (age > 60):


P(ProdromalMixedAD|SR) = P(ProdromalMixed AD|SR)P(ProdromalMixed)P(SR)=0.278

**Case 4**: Subject with an increased mitochondrial population, based on a Caloric Restriction diet type leading to abnormal fis1 and drp1 (increased) and normal mfn1, mfn2, opa1 levels within a risk group due to the age (age > 60):


P(ProdromalMixedAD|CR)=P(ProdromalMixed AD|CR)P(ProdromalMixed)P(CR) =0.614

**Case 5**: Subject within a risk group due to the age (age > 60), with problems related to a certain quality of life factors (diabetes and hypertension) but with no other evidence of abnormal biomarkers. The model calculates the probability of leading to AD due to these factors:


P(ProdromalMixedAD|quality of life factors )= P(ProdromalMixed AD|quality of life factors)P(ProdromalMixed)P(quality of life factors)=0.743

## Discussion

Mitochondrial dysfunction and oxidative stress are significant causes of neurodegeneration. Both processes produce high levels of ROS, which are detrimental to all cellular macromolecules, including nucleic acid, lipid, and protein damage (Lauritzen et al., 2016). Plenty of studies confirm that mitochondrial dysfunction is the most likely underlying mechanism of cortical contractility, especially in areas of the brain involved in learning and memory, such as the hippocampus (Stefanova et al., 2016). Mitochondrial changes can increase the production and accumulation of amyloid-β, which is directly toxic to mitochondria, and delay the neurodegenerative process. Tau hyperphosphorylation, which causes a concatenation of events, causes neurodegenerative disease, synaptic damage, and neuronal cell death, and is a detrimental feature of AD (Grimm et al., 2016). As a result, KD may provide neuroprotection by improving mitochondrial function through biochemical changes caused by inhibition of glycolysis and increased KB (ketone body) formation (Rusek et al., 2019). In addition, KB can affect mitochondrial homeostasis by altering calcium-induced membrane permeability transitions (MPTs) and preventing pores from opening.

Furthermore, certain polyunsaturated fatty acids (PUFAs) such as eicosapentaenoic acid, arachidonic acid, and docosahexaenoic acid suppress ROS production, reduce inflammatory mediators, and block voltage-gated sodium and calcium channels. In addition, it can promote neuronal cell membrane stimulation (McDonald and Cervenka, 2018). A high-fat diet is a primary etiology of overweight, obesity, and inflammation, leading to high levels of circulating free fatty acids, which stimulates the formation of amyloid and tau filaments. A high-fat diabetic diet promotes the etiology of AD, and a diet high in docosahexaenoic acid (DHA) prevents AD (Cole et al., 2010; Pugazhenthi et al., 2017).

Several lately published studies provide tools for an efficient early diagnosis or even prognosis of AD development or progression based on the Bayesian Inference or Machine Learning Techniques (Mantzavinos and Alexiou, 2017; Ali et al., 2019; Ashraf and Alexiou, 2019, 2022; De Velasco Oriol et al., 2019; Alexiou et al., 2020; Tan et al., 2021; Mirzaei and Adeli, 2022).

This article presents the first attempt in the literature to simulate the side effects of dietary types in the mitochondrial population, highly correlated to AD and other related neurodegenerative disorders. Furthermore, it provides a freely accessed programming code for reusing and expanding the experimental procedures.

In Table 2, several defined cases reveal the potential presence of Prodromal/Mixed AD due to specific biomarkers. Our base study (Putti et al., 2015) showed that HL is highly correlated to altered mitochondrial dynamics and increased ROS production. On the contrary, HFO positively affects mitochondrial functionality, reduces ROS production, and promotes mitochondrial fusion. In addition, the HFO diet can lead to decreased lipid accumulation, obesity, and insulin sensibility compared to HL (Lionetti et al., 2013; Putti et al., 2015). The results of Table 2 fit the theory of the highly correlated dietary types and mitochondrial dynamics, while mitochondrial fission and fusion seem to be depending on the high-fat diet and starvation (Table 2).

Further analysis and observational studies could also validate the model with patient data. This program is an assistive research tool for AD prognosis, but alternative prognostic tools must not replace the clinician’s diagnosis.

## Data Availability Statement

The original contributions presented in this study are included in the article/supplementary material, further inquiries can be directed to the corresponding authors.

## Author Contributions

AA and GF wrote the manuscript. AA and SC performed the analysis and data collection. SC designed the programming code. GA and MG provided the funding. MG, GA, and MK provided critical feedback and edited the manuscript. All authors revised the manuscript and agreed to its final form.

## Conflict of Interest

AA holds an unpaid position on the scientific board of the company AFNP Med Austria. The remaining authors declare that the research was conducted in the absence of any commercial or financial relationships that could be construed as a potential conflict of interest.

## Publisher’s Note

All claims expressed in this article are solely those of the authors and do not necessarily represent those of their affiliated organizations, or those of the publisher, the editors and the reviewers. Any product that may be evaluated in this article, or claim that may be made by its manufacturer, is not guaranteed or endorsed by the publisher.

## References

[B1] Afshordel S. Hagl S. Werner D. Röhner N. Kögel D. Bazan N. G. (2015). Omega-3 polyunsaturated fatty acids improve mitochondrial dysfunction in brain aging–impact of Bcl-2 and NPD-1 like metabolites. *Prostagl. Leukotr. Essent. Fatty Acids* 92 23–31. 10.1016/j.plefa.2014.05.008 24972878

[B2] Alexiou A. Chatzichronis S. Ashraf G. M. (2020). “Chapter 23 – The prediction of Alzheimer’s disease,” in *Diagnosis and Management in Dementia: The Neuroscience of Dementia*, Vol. 1, eds Martin C. R. Preedy V. R. (Amsterdam: Elsevier), 365–378. 10.1016/B978-0-12-815854-8.00023-9

[B3] Alexiou A. Mantzavinos V. D. Greig N. H. Kamal M. A. (2017). A Bayesian Model for the Prediction and Early Diagnosis of Alzheimer’s Disease. *Front. Aging Neurosci.* 9:77. 10.3389/fnagi.2017.00077 28408880 PMC5374875

[B4] Alexiou A. Nizami B. Khan F. I. Soursou G. Vairaktarakis C. Chatzichronis S. (2018a). Mitochondrial Dynamics and Proteins Related to Neurodegenerative Diseases. *Curr. Prot. Pept. Sci.* 19 850–857. 10.2174/1389203718666170810150151 28799502

[B5] Alexiou A. Soursou G. Yarla N. S. Md Ashraf G. (2018b). Proteins Commonly Linked to Autism Spectrum Disorder and Alzheimer’s Disease. *Curr. Prot. Pept. Sci.* 19 876–880. 10.2174/1389203718666170911145321 28901249

[B6] Ali M. A. Alexiou A. Ashraf G. M. (2019). “Biotechnology and Bioinformatics Applications in Alzheimer’s Disease,” in *Biological, Diagnostic and Therapeutic Advances in Alzheimer’s Disease*, eds Ashraf G. Alexiou A. (Singapore: Springer), 10.1007/978-981-13-9636-6_12

[B7] Allen B. G. Bhatia S. K. Anderson C. M. Eichenberger-Gilmore J. M. Sibenaller Z. A. Mapuskar K. A. (2014). Ketogenic diets as an adjuvant cancer therapy: history and potential mechanism. *Redox Biol.* 2 963–970. 10.1016/j.redox.2014.08.002 25460731 PMC4215472

[B8] Alzheimer’s Association (2015). Alzheimer’s Disease Facts and Figures Alzheimer’s & Dementia. *Alzheimers Dement* 11 1–88.25443857

[B9] Archer S. L. (2013). Mitochondrial dynamics—mitochondrial fission and fusion in human diseases. *New Engl. J. Med.* 369 2236–2251. 24304053 10.1056/NEJMra1215233

[B10] Ashraf G. M. Alexiou A. (2019). *Biological, Diagnostic and Therapeutic Advances in Alzheimer’s Disease - Non-Pharmacological Therapies for Alzheimer’s Disease.* New York, NY: Springer, ISBN: 978-981-13-9635-9 10.1007/978-981-13-9636-6,

[B11] Ashraf G. M. Alexiou A. (2022). *Autism Spectrum Disorder and Alzheimer’s Disease - Advances in Research.* New York, NY: Springer Nature, 10.1007/978-981-16-4558-7

[B12] Babic M. Russo G. J. Wellington A. J. Sangston R. M. Gonzalez M. Zinsmaier K. E. (2015). Miro’s N-terminal GTPase domain is required for transport of mitochondria into axons and dendrites. *J. Neurosci.* 35 5754–5771. 10.1523/JNEUROSCI.1035-14.2015 25855186 PMC4388930

[B13] Bach D. Naon D. Pich S. Soriano F. X. Vega N. Rieusset J. (2005). Expression of Mfn2, the Charcot-Marie-Tooth neuropathy type 2A gene, in human skeletal muscle: effects of type 2 diabetes, obesity, weight loss, and the regulatory role of tumor necrosis factor alpha and interleukin-6. *Diabetes* 54 2685–2693. 10.2337/diabetes.54.9.2685 16123358

[B14] Barnes D. E. Yaffe K. (2011). The projected impact of risk factor reduction on Alzheimer’s disease prevalence. *Lancet. Neurol.* 10 819–828. 10.1016/S1474-4422(11)70072-2 21775213 PMC3647614

[B15] Branco A. F. Ferreira A. Simoes R. F. Magalhães-Novais S. Zehowski C. Cope E. (2016). Ketogenic diets: from cancer to mitochondrial diseases and beyond. *Eur. J. Clin. Investig.* 46 285–298. 26782788 10.1111/eci.12591

[B16] Broom G. M. Shaw I. C. Rucklidge J. J. (2019). The ketogenic diet as a potential treatment and prevention strategy for Alzheimer’s disease. *Nutrition* 60 118–121. 30554068 10.1016/j.nut.2018.10.003

[B17] Buckley J. D. Howe P. R. (2010). Long-chain omega-3 polyunsaturated fatty acids may be beneficial for reducing obesity—a review. *Nutrients* 2 1212–1230. 10.3390/nu2121212 22254005 PMC3257626

[B18] Chen D. Li X. Zhang L. Zhu M. Gao L. (2018). A high-fat diet impairs mitochondrial biogenesis, mitochondrial dynamics, and the respiratory chain complex in rat myocardial tissues. *J. Cell. Biochem.* 119 9602–9602. 10.1002/jcb.27068 30171706 PMC6220867

[B19] Chen G. F. Xu T. H. Yan Y. Zhou Y. R. Jiang Y. Melcher K. (2017). Amyloid beta: structure, biology and structure-based therapeutic development. *Acta Pharmacol. Sin.* 38 1205–1235. 28713158 10.1038/aps.2017.28 PMC5589967

[B20] Chen H. McCaffery J. M. Chan D. C. (2007). Mitochondrial fusion protects against neurodegeneration in the cerebellum. *Cell* 130 548–562. 10.1016/j.cell.2007.06.026 17693261

[B21] Cho B. Choi S. Y. Cho H. M. Kim H. J. Sun W. (2013). Physiological and pathological significance of dynamin-related protein 1 (drp1)-dependent mitochondrial fission in the nervous system. *Exp. Neurob.* 22:149. 10.5607/en.2013.22.3.149 24167410 PMC3807002

[B22] Cho D. H. Nakamura T. Fang J. Cieplak P. Godzik A. Gu Z. (2009). S-nitrosylation of Drp1 mediates beta-amyloidrelated mitochondrial fission and neuronal injury. *Science* 324 102–105. 19342591 10.1126/science.1171091 PMC2823371

[B23] Christen Y. (2000). Oxidative stress and Alzheimer disease. *Am. J. Clin. Nutr.* 71 621S–629S. 10.1093/ajcn/71.2.621s 10681270

[B24] Cole G. M. Ma Q. L. Frautschy S. A. (2010). Dietary fatty acids and the aging brain. *Nutrit. Rev.* 68(Suppl._2), S102–S111. 10.1111/j.1753-4887.2010.00345.x 21091943 PMC4019000

[B25] De Velasco Oriol J. Vallejo E. E. Estrada K. Gerardo J. (2019). Benchmarking machine learning models for late-onset alzheimer’s disease prediction from genomic data. *BMC Bioinform.* 20:709. 10.1186/s12859-019-3158-x 31842725 PMC6915925

[B26] DeFronzo R. A. Tripathy D. (2009). Skeletal muscle insulin resistance is the primary defect in type 2 diabetes. *Diabetes Care* 32 157–163. 10.2337/dc09-S302 PMC2811436 19875544

[B27] Dietrich M. O. Liu Z. W. Horvath T. L. (2013). Mitochondrial dynamics controlled by mitofusins regulate Agrp neuronal activity and diet-induced obesity. *Cell* 155 188–199. 10.1016/j.cell.2013.09.004 24074868 PMC4142434

[B28] DuBoff B. Gotz J. Feany M. B. (2012). Tau promotes neurodegeneration via Drp1 mislocalization in vivo. *Neuron* 75 618–632. 10.1016/j.neuron.2012.06.026 22920254 PMC3428596

[B29] Dubois B. Feldman H. H. Jacova C. Dekosky S. T. Barberger-Gateau P. Cummings J. (2007). Research criteria for the diagnosis of Alzheimer’s disease: revising the NINCDS-ADRDA criteria. *Lancet Neurol.* 6 734–746. 10.1016/S1474-4422(07)70178-3 17616482

[B30] Figlewicz D. P. Jay J. L. Acheson M. A. Magrisso I. J. West C. H. Zavosh A. (2013). Moderate high fat diet increases sucrose self-administration in young rats. *Appetite* 61 19–29. 10.1016/j.appet.2012.09.021 23023044 PMC3538965

[B31] Fischer H. P. (2008). Mathematical modeling of complex biological systems: from parts lists to understanding systems behavior. *Alcohol. Res. Health* 31 49–59. 23584751 PMC3860444

[B32] Gammone M. A. Riccioni G. Parrinello G. D’Orazio N. (2019). Omega-3 polyunsaturated fatty acids: benefits and endpoints in sport. *Nutrients* 11:46. 10.3390/nu11010046 PMC6357022 30591639

[B33] Gao J. Wang L. Liu J. Xie F. Su B. Wang X. (2017). Abnormalities of mitochondrial dynamics in neurodegenerative diseases. *Antioxidants* 6:25. 10.3390/antiox6020025 PMC5488005 28379197

[B34] Grimm A. Eckert A. (2017). Brain aging and neurodegeneration: from a mitochondrial point of view. *J. Neurochem.* 143 418–431. 28397282 10.1111/jnc.14037 PMC5724505

[B35] Grimm A. Friedland K. Eckert A. (2016). Mitochondrial dysfunction: the missing link between aging and sporadic Alzheimer’s disease. *Biogerontology* 17 281–296. 26468143 10.1007/s10522-015-9618-4

[B36] Griparic L. van der Wel N. N. Orozco I. J. Peters P. J. van der Bliek A. M. (2004). Loss of the intermembrane space protein mgm1/opa1 induces swelling and localized constrictions along the lengths of mitochondria. *J. Biol. Chem.* 279 18792–18798. 10.1074/jbc.M400920200 14970223

[B37] Hernández-Alvarez M. I. Thabit H. Burns N. Shah S. Brema I. Hatunic M. (2010). Subjects with early-onset type 2 diabetes show defective activation of the skeletal muscle PGC-1α/Mitofusin-2 regulatory pathway in response to physical activity. *Diabetes Care* 33 645–651. 10.2337/dc09-1305 20032281 PMC2827524

[B38] Højsgaard S. (2012). Graphical independence networks with the gRain package for R. *J Stat Softw.* 46 1–26. 10.18637/jss.v046.i1022837731

[B39] Huang Y. Mucke L. (2012). Alzheimer mechanisms and therapeutic strategies. *Cell* 148 1204–1222. 22424230 10.1016/j.cell.2012.02.040 PMC3319071

[B40] Israeli-Korn S. D. Masarwa M. Schechtman E. Abuful A. Strugatsky R. Avni S. (2010). Hypertension increases the probability of Alzheimer’s disease and of mild cognitive impairment in an Arab community in northern Israel. *Neuroepidemiology* 34 99–105. 10.1159/000264828 20016220 PMC2855875

[B41] Itoh K. Nakamura K. Iijima M. Sesaki H. (2013). Mitochondrial dynamics in neurodegeneration. *Trends Cell Biol.* 23 64–71. 23159640 10.1016/j.tcb.2012.10.006 PMC3558617

[B42] Jheng H. F. Tsai P. J. Guo S. M. Kuo L. H. Chang C. S. Su I. J. (2012). Mitochondrial fission contributes to mitochondrial dysfunction and insulin resistance in skeletal muscle. *Mol. Cell. Biol.* 32 309–319. 10.1128/MCB.05603-11 22083962 PMC3255771

[B43] Kageyama Y. Zhang Z. Roda R. Fukaya M. Wakabayashi J. Wakabayashi N. (2012). Mitochondrial division ensures the survival of postmitotic neurons by suppressing oxidative damage. *J. Cell Biol.* 197 535–551. 10.1083/jcb.201110034 22564413 PMC3352955

[B44] Lalia A. Z. Lanza I. R. (2016). Insulin-Sensitizing Effects of Omega-3 Fatty Acids: lost in Translation? *Nutrients* 8:329. 10.3390/nu8060329 27258299 PMC4924170

[B45] Lauritzen K. H. Hasan-Olive M. M. Regnell C. E. Kleppa L. Scheibye-Knudsen M. Gjedde A. (2016). A ketogenic diet accelerates neurodegeneration in mice with induced mitochondrial DNA toxicity in the forebrain. *Neurobiol. Aging* 48 34–47. 10.1016/j.neurobiolaging.2016.08.005 27639119 PMC5629920

[B46] Lepretti M. Martucciello S. Burgos Aceves M. A. Putti R. Lionetti L. (2018). Omega-3 fatty acids and insulin resistance: focus on the regulation of mitochondria and endoplasmic reticulum stress. *Nutrients* 10:350. 10.3390/nu10030350 29538286 PMC5872768

[B47] Li X. C. Hu Y. Wang Z. H. Luo Y. Zhang Y. Liu X. P. (2016). Human wild-type full-length tau accumulation disrupts mitochondrial dynamics and the functions via increasing mitofusins. *Sci. Rep.* 6:24756. 10.1038/srep24756 27099072 PMC4838862

[B48] Liao C. Ashley N. Diot A. Morten K. Phadwal K. Williams A. (2017). Dysregulated mitophagy and mitochondrial organization in optic atrophy due to OPA1 mutations. *Neurology* 88 131–142. 10.1212/WNL.0000000000003491 27974645 PMC5224718

[B49] Lima P. A. Sampaio L. P. Damasceno N. R. (2014). Neurobiochemical mechanisms of a ketogenic diet in refractory epilepsy. *Clinics* 69 699–705. 10.6061/clinics/2014(10)09 25518023 PMC4221309

[B50] Lionetti L. Mollica M. P. Donizzetti I. Gifuni G. Sica R. Pignalosa A. (2014). High-lard and high-fish-oil diets differ in their effects on function and dynamic behaviour of rat hepatic mitochondria. *PLoS One* 9:e92753. 10.1371/journal.pone.0092753 24663492 PMC3963938

[B51] Lionetti L. Sica R. Mollica M. P. Putti R. (2013). High-lard and high-fish oil diets differ in their effects on insulin resistance development, mitochondrial morphology and dynamic behaviour in rat skeletal muscle. *Food Nutr. Sci.* 4 105–112. 10.4236/fns.2013.49A1017

[B52] Magrané J. Cortez C. Gan W. B. Manfredi G. (2014). Abnormal mitochondrial transport and morphology are common pathological denominators in SOD1 and TDP43 ALS mouse models. *Hum. Mole. Genet.* 23 1413–1424. 10.1093/hmg/ddt528 24154542 PMC3929084

[B53] Manczak M. Reddy P. H. (2012). Abnormal interaction between the mitochondrial fission protein Drp1 and hyperphosphorylated tau in Alzheimer’s disease neurons: implications for mitochondrial dysfunction and neuronal damage. *Hum. Mole. Genet.* 21 2538–2547. 10.1093/hmg/dds072 22367970 PMC3349426

[B54] Mantzavinos V. Alexiou A. (2017). Biomarkers for Alzheimer’s Disease Diagnosis. *Curr. Alzheimer Res.* 14 1149–1154. 28164766 10.2174/1567205014666170203125942 PMC5684784

[B55] Mattson M. P. (2004). Pathways towards and away from Alzheimer’s disease. *Nature* 430 631–639. 15295589 10.1038/nature02621 PMC3091392

[B56] McDonald T. J. Cervenka M. C. (2018). Ketogenic diets for adult neurological disorders. *Neurotherapeutics* 15 1018–1031. 30225789 10.1007/s13311-018-0666-8 PMC6277302

[B57] Mirzaei G. Adeli H. (2022). Machine learning techniques for diagnosis of alzheimer disease, mild cognitive disorder, and other types of dementia. *Biomed. Sign. Proc. Control* 72:103293. 10.1016/j.bspc.2021.103293

[B58] Mishra P. Chan D. C. (2016). Metabolic regulation of mitochondrial dynamics. *J. Cell Biol.* 212 379–387. 26858267 10.1083/jcb.201511036 PMC4754720

[B59] Modrego P. J. Ferrández J. (2004). Depression in Patients with Mild cognitive impairment increases the risk of developing dementia of Alzheimer typea prospective cohort study. *Arch. Neurol.* 61 1290–1293. 10.1001/archneur.61.8.1290 15313849

[B60] Natarajan V. Chawla R. Mah T. Vivekanandan R. Tan S. Y. Sato P. Y. (2020). Mitochondrial dysfunction in age-related metabolic disorders. *Proteomics* 20:1800404. 10.1002/pmic.201800404 PMC12087358 32131138

[B61] Ni H. M. Williams J. A. Ding W. X. (2015). Mitochondrial dynamics and mitochondrial quality control. *Redox Biol.* 4 6–13. 10.1016/j.redox.2014.11.006 25479550 PMC4309858

[B62] Pagani L. Eckert A. (2011). Amyloid-Beta interaction with mitochondria. *Internat. J. Alzheimer’s Dis.* 2011:925050. 10.4061/2011/925050 21461357 PMC3065051

[B63] Pugazhenthi S. Qin L. Reddy P. H. (2017). Common neurodegenerative pathways in obesity, diabetes, and Alzheimer’s disease. *Biochimica et biophysica acta* 1863 1037–1045. 27156888 10.1016/j.bbadis.2016.04.017 PMC5344771

[B64] Putti R. Sica R. Migliaccio V. Lionetti L. (2015). Diet impact on mitochondrial bioenergetics and dynamics. *Front. Physiol.* 6:109. 10.3389/fphys.2015.00109 25904870 PMC4389347

[B65] Rusek M. Pluta R. Ułamek-Kozioł M. Czuczwar S. J. (2019). Ketogenic diet in Alzheimer’s disease. *Internat. J. Mole. Sci.* 20:3892. 10.3390/ijms20163892 31405021 PMC6720297

[B66] Saada A. (2014). Mitochondria: mitochondrial OXPHOS (dys) function ex vivo–The use of primary fibroblasts. *Internat. J. Biochem. Cell Biol.* 48 60–65. 10.1016/j.biocel.2013.12.010 24412346

[B67] Sheng Z. H. Cai Q. (2012). Mitochondrial transport in neurons: impact on synaptic homeostasis and neurodegeneration. *Nat. Rev. Neurosci.* 13 77–93. 22218207 10.1038/nrn3156 PMC4962561

[B68] Stefanova N. A. Muraleva N. A. Maksimova K. Y. Rudnitskaya E. A. Kiseleva E. Telegina D. V. (2016). An antioxidant specifically targeting mitochondria delays progression of Alzheimer’s disease-like pathology. *Aging* 8:2713. 10.18632/aging.101054 27750209 PMC5191865

[B69] Suárez-Rivero J. M. Villanueva-Paz M. la Cruz-Ojeda D. De la Mata M. Cotán D. Oropesa-Avila M. (2017). Mitochondrial dynamics in mitochondrial diseases. *Diseases* 5:1. 10.3390/diseases5010001 PMC5456341 28933354

[B70] Tan M. S. Cheah P. L. Chin A. V. Looi L. M. Chang S. W. (2021). A review on omics-based biomarkers discovery for Alzheimer’s disease from the bioinformatics perspectives: statistical approach vs machine learning approach. *Comp. Biol. Med.* 139:104947. 10.1016/j.compbiomed.2021.104947 34678481

[B71] Tenenbaum J. (1998). Bayesian modeling of human concept learning. *Adv. Neural. Inform. Proc. Syst.* 1998:11.

[B72] Twig G. Hyde B. Shirihai O. S. (2008). Mitochondrial fusion, fission and autophagy as a quality control axis: the bioenergetic view. *Biochimica et Biophysica Acta* 1777 1092–1097. 10.1016/j.bbabio.2008.05.001 18519024 PMC3809017

[B73] Tzoufras I. (2009). *Bayesian Modeling UsingWinb.* ugs. Hoboken, NJ: Wiley.

[B74] Vidakovic B. (2011). *Statistics for Bioengineering Sciences.* New York, NY: Springer.

[B75] Wai T. Langer T. (2016). Mitochondrial dynamics and metabolic regulation. *Trends Endocrinol. Metabol.* 27 105–117. 10.1016/j.tem.2015.12.001 26754340

[B76] Wang X. Su B. Lee H. G. Li X. Perry G. Smith M. A. (2009). Impaired balance of mitochondrial fission and fusion in Alzheimer’s disease. *J. Neurosci.* 29 9090–9103. 10.1523/JNEUROSCI.1357-09.2009 19605646 PMC2735241

[B77] Züchner S. Mersiyanova I. V. Muglia M. Bissar-Tadmouri N. Rochelle J. Dadali E. L. (2004). Mutations in the mitochondrial GTPase mitofusin 2 cause Charcot-Marie-Tooth neuropathy type 2A. *Nat. Genet.* 36 449–451. 10.1038/ng1341 15064763

